# Integrated UPLC/Q-TOF-MS/MS Analysis and Network Pharmacology to Reveal the Neuroprotective Mechanisms and Potential Pharmacological Ingredients of Aurantii Fructus Immaturus and Aurantii Fructus

**DOI:** 10.3390/ph17020239

**Published:** 2024-02-12

**Authors:** Mingyang Qiu, Jianqing Zhang, Wenlong Wei, Yan Zhang, Mengmeng Li, Yuxin Bai, Hanze Wang, Qian Meng, De-an Guo

**Affiliations:** 1 National Engineering Research Center of TCM Standardization Technology, Shanghai Institute of Materia Medica, Chinese Academy of Sciences, Shanghai 200100, China; qiumingyang2022@163.com (M.Q.);; 2 College of Pharmacy, Changchun University of Chinese Medicine, Changchun 130117, China

**Keywords:** UPLC/Q-TOF-MS/MS, Aurantii Fructus Immaturus, Aurantii Fructus, network pharmacology, neuroprotection

## Abstract

Aurantii Fructus (AF) and Aurantii Fructus Immaturus (AFI) have been used for thousands of years as traditional Chinese medicine (TCM) with sedative effects. Modern studies have shown that Citrus plants also have protective effects on the nervous system. However, the effective substances and mechanisms of action in Citrus TCMs still remain unclear. In order to explore the pharmacodynamic profiles of identified substances and the action mechanism of these herbs, a comprehensive approach combining ultra-high-performance liquid chromatography with quadrupole time-of-flight mass spectrometry (UPLC/Q-TOF-MS/MS) analysis and network pharmacology was employed. Firstly, UNIFI 2.1.1 software was used to identify the chemical characteristics of AF and AFI. Secondly, the SwissTargetPrediction database was used to predict the targets of chemical components in AF and AFI. Targets for neuroprotection were also collected from GeneCards: The Human Gene Database (GeneCards-Human Genes|Gene Database|Gene Search). The networks between targets and compounds or diseases were then constructed using Cytoscape 3.9.1. Finally, the Annotation, Visualization and Integrated Discovery Database (DAVID) (DAVID Functional Annotation Bioinformatics Microarray Analysis) was used for GO and pathway enrichment analysis. The results showed that 50 of 188 compounds in AF and AFI may have neuroprotective biological activities. These activities are associated with the regulatory effects of related components on 146 important signaling pathways, derived from the KEGG (KEGG: Kyoto Encyclopedia of Genes and Genomes), such as neurodegeneration (hsa05022), the Alzheimer’s disease pathway (hsa05010), the NF-kappa B signaling pathway (hsa04064), the hypoxia-inducible factor (HIF)-1 signaling pathway (hsa04066), apoptosis (hsa04210), the epidermal growth factor receptor (EGFR) tyrosine kinase inhibitor resistance signaling pathway (hsa01521), and others, by targeting 108 proteins, including xanthine dehydrogenase (XDH), glutamate ionotropic receptor NMDA type subunit 2B (GRIN2B), and glucose-6-phosphate dehydrogenase (G6PD), among others. These targets are thought to be related to inflammation, neural function and cell growth.

## 1. Introduction

Aurantii Fructus (AF) and Aurantii Fructus Immaturus (AFI) have been used in traditional Chinese medicine (TCM) for thousands of years [1]. AF and AFI are the fruits of *Citrus aurantium* L. (CA) (bitter orange) and their cultivated varieties [2]. And *Citrus aurantium* L. Cv. *daidai* (CAD) is the most commonly used cultivated variety of *Citrus aurantium* L. and is widely grown as a medicinal plant [3]. AF and AFI are collected at different stages of fruit growth with diverse clinical efficacy; the effect of AFI on promoting qi is obviously better than that of AF, and they are thus are recorded in the Chinese Pharmacopoeia as two distinct medicinal materials [4]. According to TCM theory, AF and AFI each have their own unique clinical applications [5]. Although AF and AFI have common effects of regulating visceral functions [6], AF is always used to alleviate chest pain and improve gastrointestinal functions, such as alleviating dyspepsia in a gentle yet efficient manner [7]. AFI, compared to AF, expresses a more rapid and robust method of action and is often employed to disperse severe abdominal distention and to eliminate phlegm [8]. We found that Citrus plants, including *Citrus aurantium* L., have beneficial effects on those with neurodegenerative diseases [9], suggesting AF and AFI to have potential protective effects on the nervous system. Therefore, it is reasonable to explore the protective effects of AF and AFI on nervous system. Currently, excitotoxicity and oxidative stress are recognized as two important aspects of nervous system damage [10]. Hence, we believe that it is meaningful to study the chemical components related to excitotoxicity and oxidative stress in AF and AFI. At present, chemical analysis methods, including chromatography [11], nuclear magnetic resonance (NMR) spectroscopy [12], and mass spectrometry (MS) [13], are usually used to study the chemical constituents of plant drugs. Among them, ultra-high-performance liquid chromatography (UPLC) alongside high-resolution mass spectrometry (HR-MS) can simultaneously detect a variety of chemical components in plant drugs [14]; however, to obtain accurate identification results, the UPLC-HR-MS detection results must be compared with the standard chromatogram of chemical components or the mass spectrometry database [15]. As an auxiliary mass spectrum analysis software, UNIFI supports multi-user, server-based workgroups to complete liquid chromatography (LC), LC/MS, and LC/MS/MS data collection, storage, management, mining, and sharing, which can greatly improve collaboration efficiency [16,17].

In this study, the chemical compositions of AF and AFI derived from *Citrus aurantium* L. and *Citrus aurantium* L. Cv. *daidai* were systematically evaluated with UNIFI software with UPLC/quadrupole time-of-flight (Q-TOF)-MS/MS. The chemical similarities and differences between AF and AFI were summarized. Furthermore, the target of compounds and the target of neuroprotection were predicted using the method of network pharmacology [18]. Finally, identifying bioactive compounds, potential targets, and signaling pathways relevant to the neuroprotection with AF and AFI was realized using an integrative network analysis [19].

The results indicated that 50 of the 188 compounds in AF and AFI may be bioactive, which may be related to their targeting of 108 targets such as XDH, GRIN2B, AKT1, PRKCG, CAPN1, CSNK2A1, G6PD, etc. One hundred and forty-six important signaling pathways were identified, including neurodegeneration (hsa05022), the Alzheimer’s disease pathway (hsa05010), the NF-kappa B signaling pathway (hsa04064), the HIF-1 signaling pathway (hsa04066), apoptosis (hsa04210), and the EGFR tyrosine kinase inhibitor resistance signaling pathway (hsa01521), etc. 

## 2. Results and Discussion

### 2.1. Identification of Compounds in AF and AFI

**Figure 1:**
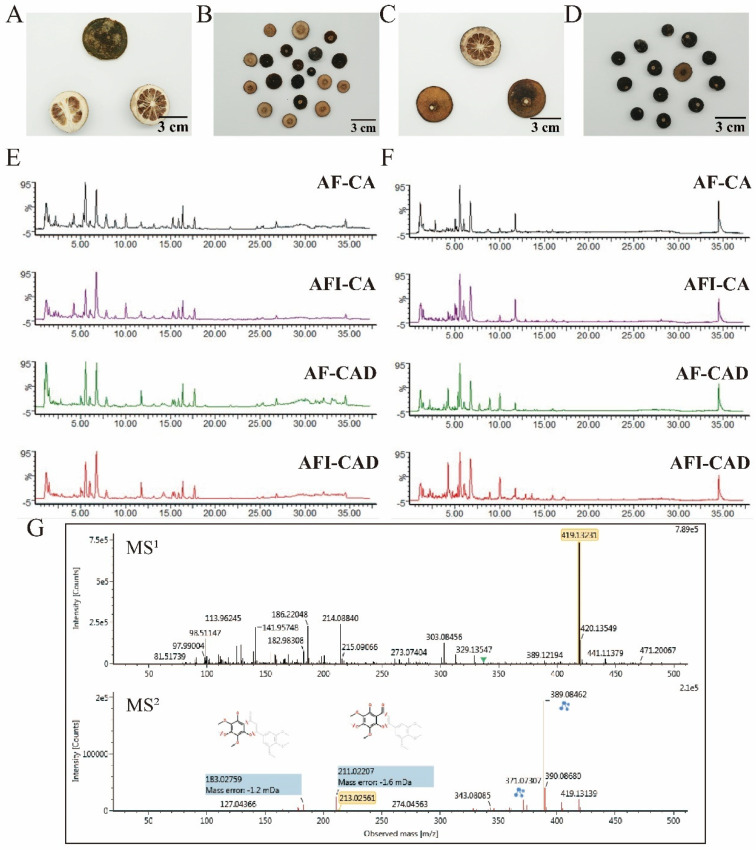
Identification of compounds in AF and AFI. (**A**) AF-CA; (**B**) AFI-CA; (**C**) AF-CAD; (**D**) AFI-CAD; (**E**) total ion chromatography of samples in positive ion mode; (**F**) total ion chromatography of samples in negative ion mode; (**G**) the identification process of compounds in UNIFI software: No. 142 compound identified as Gardenin A.

The total ion chromatograms of AF and AFI in both positive and negative ion modes are presented in Figure 1A–F. The process of identifying compounds via UNIFI software is shown in Figure 1G. The retention times and the MS data of the characterized compounds are summarized in Table 1. A total of 188 compounds were identified by UNIFI software based on the self-built database. Among these compounds, compounds (**46**, **47**, **92**, **106**, **119**, **130**, **154**) were unambiguously identified by comparison with reference compounds.

### 2.2. Identification of the AFI- and AF-Associated Targets and Analysis of the “Compound–Target” Network

**Figure 2:**
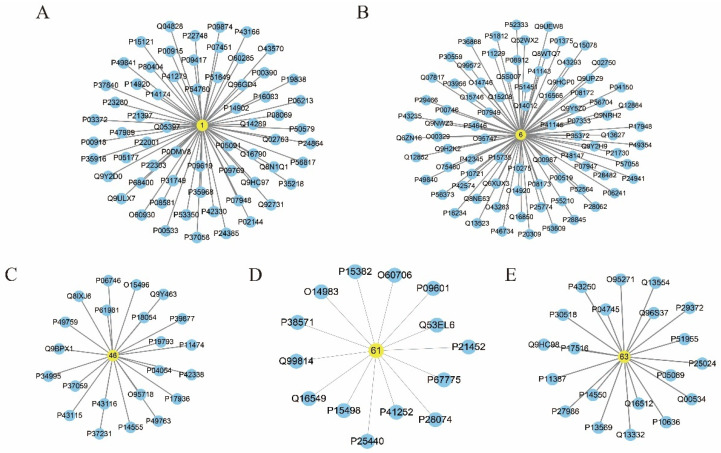
Compound–target networks for AFI and AF. (**A**) Compound **1** (7-Hydroxycoumarin) compound–target network; (**B**) Compound **6** (Limonin) compound–target network; (**C**) Compound **46** ((+/−)-Naringenin) compound–target network; (**D**) Compound **61** (Helenalin) compound–target network; (**E**) Compound **63** (Kaempferol) compound–target network.

Using the SwissTargetPrediction databases, we obtained the 9021 target proteins of the 188 compounds in AFI and AF. The entire list of targets of each compound is provided in Supplementary Table S2. After removing redundancy, we identified 1052 AFI- or AF-associated targets (Supplementary Table S3). Compound–target networks were constructed on the basis of compounds **1** (7-Hydroxycoumarin), **6** (Limonin), **46** ((+/−)-Naringenin), **61** (Helenalin), and **63** (Kaempferol) and their corresponding targets, as shown in Figure 2. The round, yellow nodes and round, blue nodes represent the compounds and targets, respectively, and the edges represent the interactions between compounds and targets.

### 2.3. Identification of the Neuroprotective Targets and Analysis of the “Disease–Target” Network

**Figure 3:**
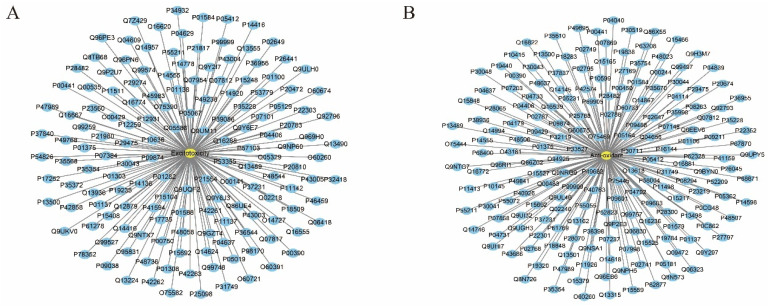
Disease–target networks for neuroprotection. (**A**) Excitotoxicity-associated target network; (**B**) antioxidation target network.

By means of the available resource, namely, the GeneCards: The Human Gene Database. we obtained 151 excitotoxicity-associated targets (relevance > 1.0) and 187 antioxidant-associated targets (relevance > 1.0). And detailed information on the collected targets is provided in Supplementary Table S4 (excitotoxicity-associated targets) and Supplementary Table S5 (antioxidant-associated). Disease–target networks were constructed, as shown in Figure 3. The network consisted of two parts (A: an excitotoxicity-associated target network with 151 nodes; B: an antioxidation target network with 187 nodes). The round, blue nodes and round, yellow nodes represent the targets and diseases, respectively, and the edges represent the interactions between diseases and targets.

### 2.4. Recognition of the Candidate Compounds and Potential Targets and Analysis of the “Compound–Disease–Target” Network

**Figure 4:**
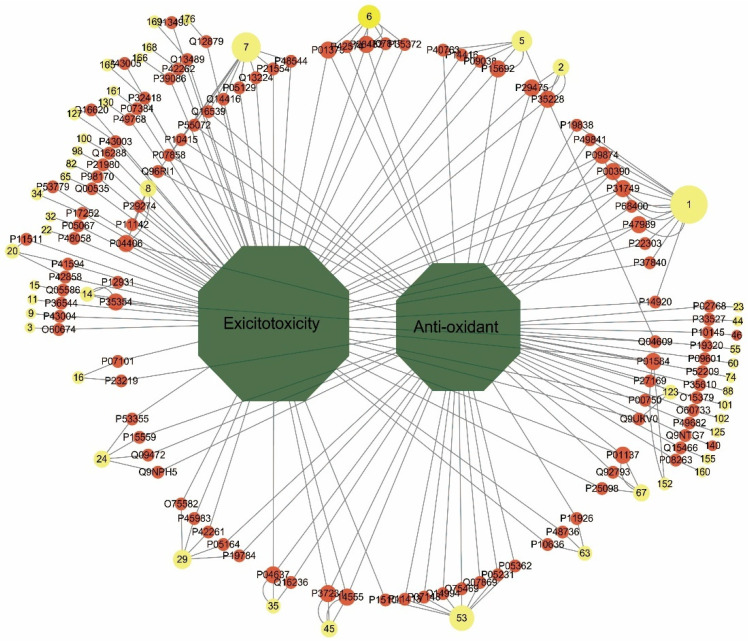
Compound–disease–target network. The yellow, red, and green nodes represent the compounds (the numbers represent the serial numbers of the compounds in Table 1), targets and diseases, respectively, and a node’s size is proportional to its degree. The edges represent the interactions between any two nodes.

**Figure 5:**
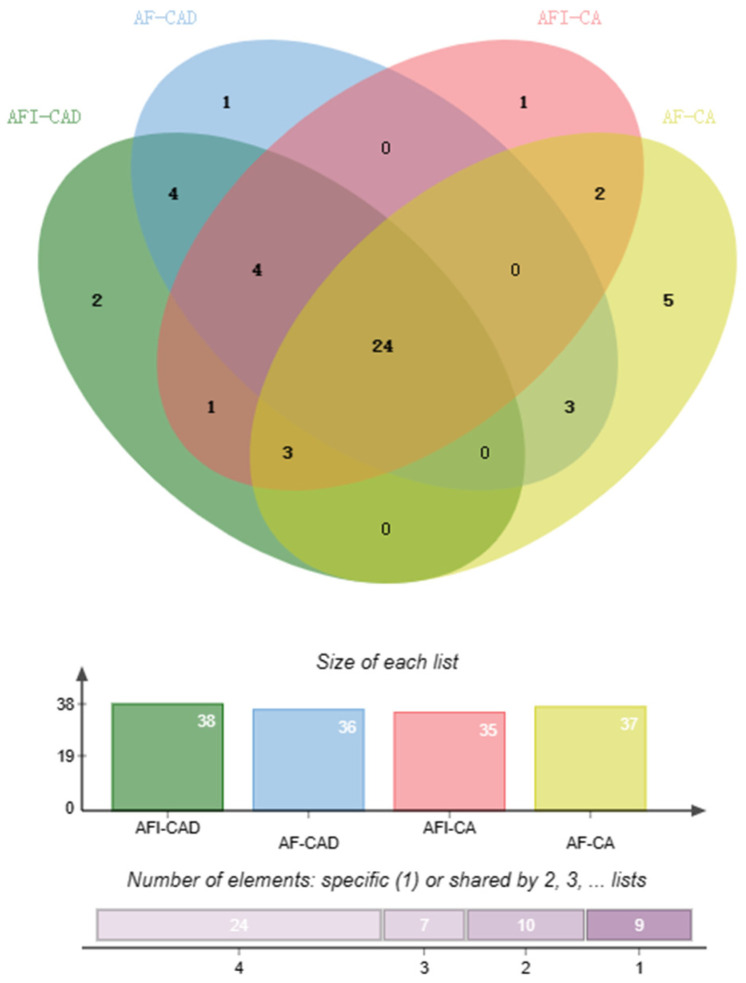
A Venn diagram of neuroprotective candidate compounds among AF-CA, AFI-CA, AF-CAD, and AFI-CAD.

A total of 125 overlapping protein targets were recognized, and 50 candidate compounds were obtained, as described in Supplementary Table S6. Figure 4 shows the compound–disease–target network, which was composed of one hundred and seventy-seven nodes (one hundred and twenty-five targets, fifty compounds, and two diseases) and two hundred and fifty edges. The round, yellow nodes, round, red nodes, and green nodes represent the compounds, targets, and diseases, respectively, and each node size is proportional to its degree. The edges represent the interactions between any two types of nodes. The results showed that the 50 compounds and 125 targets may be the candidate bio-active substances and the potential pharmacological targets for neuroprotection of AF and AFI. In particular, the neuroprotective candidate compounds are shown in Table 2 and Figure 5, and the potential pharmacological targets are shown in Table 3. There are significant differences in the chemical composition of AF and AFI [2], and we found that the neuroprotective effects of the compounds of AF and AFI are less different, as shown in Figure 5. Limonin in Table 2 is present in four samples, and studies have shown that it has a neuroprotective effect [20].

### 2.5. GO and Pathway Enrichment Analyses of Potential Targets

**Figure 6:**
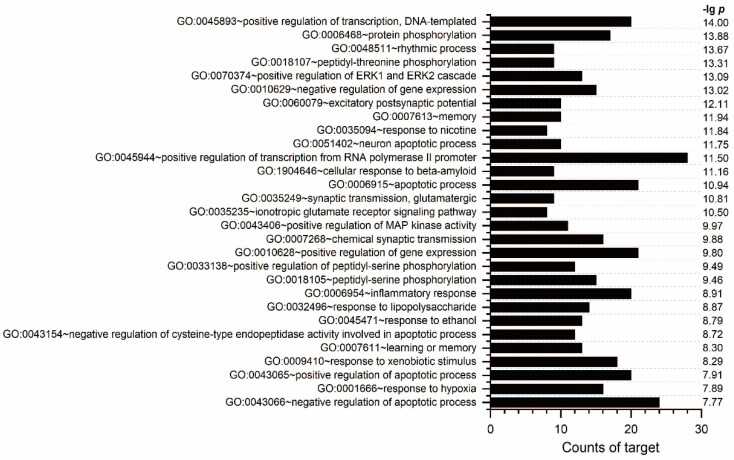
The top 30 enriched gene ontology terms for the biological processes of potential targets.

One of the functions of GO processes is to predict genes related to a disease [21]. GO and pathway enrichment analyses of the 108 potential targets for neuroprotection in AF and AFI were performed using the DAVID database to understand the relationships between functional units and their underlying significance in the biological system networks [22]. All of the biological processes and pathways were extracted (*p* ≤ 0.05). Figure 6 lists the top 30 most significantly enriched GOBP terms. Supplementary Tables S7 and S8 provide detailed information about the biological processes and signaling pathways. In total, 146 related pathways were identified, including pathways of neurodegeneration (hsa05022), the Alzheimer’s disease pathway (hsa05010), the NF-kappa B signaling pathway (hsa04064), the HIF-1 signaling pathway (hsa04066), apoptosis (hsa04210), and the EGFR tyrosine kinase inhibitor resistance signaling pathway (hsa01521). And numerous targets were involved in the memory process, gene expression, the rhythmic process, the neuron apoptotic process, and the apoptotic process.

## 3. Materials and Methods

### 3.1. Experimental Compounds Discovery

#### 3.1.1. Chemicals and Materials

AF-CA and AFI-CA (batch number: S202108-0932, S202101-0929) were collected from Xinyu County, Jiangxi Province, China. AF-CAD and AFI-CAD (batch number: S202108-0933, S202106-0930) were collected from Jinhua County, Zhejiang Province, China. And all samples were stored at room temperature until experimentation. All collected samples have accompanying voucher specimens held in the National Engineering Research Center of TCM Standardization Technology, Shanghai Institute of Materia Medica (Shanghai Institute of Material Medical Chinese Academy of Sciences (cas.cn)) accessed on 6 February 2024), Chinese Academy of Sciences, Shanghai, China.

Seven compounds were used as reference standards (purity > 98%): namely, Hesperidins, Nobiletin, Tangeretin, Didymin, Naringin, Naringenin, and Narirutin, which were purchased from Shanghai Standard Technology Co. Ltd. (Shanghai, China) (nature-standard.com). Ultra-pure water was prepared by a Milli-Q water purification system (Millipore, Bedford, MA, USA). All other chemicals were of analytical grade and obtained commercially. All extractions used in UPLC-Q-TOF were carried out with high-performance liquid chromatography (HPLC)-grade solvents.

#### 3.1.2. Sample Preparation

AF-CA, AFI-CA, AF-CAD, and AFI-CAD powder (100 mg) were extracted successively with 2 mL of 50% MeOH in an ultrasonic bath (40 kHz) for 30 min. After centrifuging at 15,890× *g* for 10 min, the supernatant was used for later analysis.

#### 3.1.3. UPLC/Q-TOF-MS/MS Analysis

The equipment used was an ACQUITY UPLC I-Class System coupled to a Xevo G2–XS Q-TOF mass spectrometer (Waters, Milford, MA, USA). Each prepared sample was subjected to LC-MS/MS analysis with a scan event recording MS/MS spectrum in data-dependent acquisition mode. An ACQUITY UPLC^®^ BEH C18 (1.7 µm × 2.1 × 100 mm) column was used for the separation of analytes in the extracts with a flow rate of 0.2 mL/min at 30 °C. The injection volume was 2 μL. A linear gradient program with a mobile phase system including solvent A (0.1% formic acid in water, *v*/*v*) and solvent B (0.1% formic acid in acetonitrile, *v*/*v*) was performed as follows: solvent A at 85~79% for 0.01~3 min, 79% for 3~7 min, 79~65% for 7~12 min, 65~50% for 12~16 min, 50~40% for 16~22 min, 40~20% for 22~25 min, and 20~5% for 25~29 min, with isocratic elution performed at 5% for 4 min. The MS spectra were acquired in positive and negative ion modes to provide complementary information for structural identification. The scan range was from 100 to 1200 m/z. The acquisition parameters for Q-TOF mass spectra were as follows: cone voltage at 40 V for both electron spray ionization (ESI)+ and ESI− modes. The desolvation gas was set to 800 L/h at a temperature of 300 °C, the cone gas was set to 50 L/h, and the source temperature was set to 120 °C. The mass spectrometry was operated linearly in data-dependent acquisition mode at a low energy level of 25–35 eV and a high energy level of 40–50 eV. All analyses were acquired using the LockSpray to ensure accuracy and reproducibility. Leucine-enkephalin was used as the lock mass at a concentration of 300 ng/mL and flow rate of 20 μL/min. Data were collected in continuum mode, the LockSpray interval was set at 10 s. The data acquisition rate was set to 1.5 s. All acquisition of data was controlled by Waters Masslynx v4.2 software (Waters, Manchester, UK).

#### 3.1.4. UNIFI Data Processing Method

The chemical constituent library of AF and AFI was firstly established for component analysis [23]: The complete information on the compounds of AF and AFI was collected and obtained by searching the China National Knowledge Infrastructure (CNKI) (cnki.net, accessed on 31 January 2024), PubMed (PubMed (nih.gov) accessed on 31 January 2024), PubChem (PubChem (nih.gov) accessed on 6 February 2024), Traditional Chinese Medicine Systems Pharmacology Database and Analysis Platform (TCMSP) (tcmsp-e.com, accessed on 31 January 2024), ChemSpider (chemspider.com, accessed on 31 January 2024), and other databases. The self-built compound library, including compound name and chemical structure (saved in “mol” format), was imported into UNIFI. Among them, a total of 1190 compounds were listed (Supplement Table S1). We imported the original files on the samples solution and blank sample solution obtained by UPLC-Q-TOF-MS into the UNIFI software for sample comparison. Based on the automatic matching function of the UNIFI software, compounds can be quickly identified. The parameter settings were as follows: analysis time range, 1–36 min; quality allowable error range, ±10 ppm; quality testing range, 100 *m*/*z* to 1200 *m*/*z*; positive adducts including H^+^, Na^+^, and K^+^; and negative adducts containing H^−^, HCOO^−^, and Cl^−^. Finally, using the MassLynx workstation, the above identification results were reviewed in combination with the precise mass of excimer ions, retention time, fragment ion information, and the literature [17].

### 3.2. Target Prediction of the Compounds in AFI and AF and Neuroprotective Target Collection

#### 3.2.1. Predicting Targets of Compounds in AFI and AF

According to our study (Section 3.1), all of the compounds in AFI and AF were chosen to predict the biological targets. The canonical SMILES [24] of the compounds were uploaded into the SwissTargetPrediction database (http://www.swisstargetprediction.ch/ accessed on 31 January 2024) to obtain the UniProt IDs for predicting targets [25].

#### 3.2.2. Collecting Neuroprotective Targets

“Excitotoxicity” and “antioxidation” are considered to be the two key directions of neuroprotection [26]. The biological targets related to neuroprotection were selected from the GeneCards: The Human Gene Database [27] (https://www.genecards.org/, accessed on 6 February 2024, version 5.15.0, relevance > 1.0) using “excitotoxicity” and “antioxidation” as keywords [28].

### 3.3. Identification of Potential Targets for the Neuroprotection of AFI and AF

#### 3.3.1. Screening Candidate Compounds and Potential Targets

We selected the overlapping targets of AF and AFI for neuroprotection and used the compounds corresponding to these targets as candidate compounds.

#### 3.3.2. Gene Ontology (GO) and Pathway Enrichment of Potential Targets

The Gene Ontology (GO) biological process (BP) was analyzed to further validate whether the potential targets were indeed matched for neuroprotection [29]. GO and Kyoto Encyclopedia of Genes and Genomes (KEGG) [30] signaling pathway analyses were carried out using the Database for Annotation, Visualization and Integrated Discovery (DAVID) (https://david.ncifcrf.gov/, accessed on 31 January 2024, version v2023q1). A *p*-value ≤ 0.05 was considered significant.

#### 3.3.3. Constructing the Network of Compounds, Diseases, and Targets

To comprehensively understand the neuroprotection of AF and AFI, the compound–target and disease–target networks were constructed using Cytoscape 3.9.1 (Bethesda, MD, USA) [31]. In these networks, the nodes represented the compounds, diseases, targets, or signaling pathways, and the edges represented their interactions [32].

## 4. Conclusions

In this study, a comprehensive method combining UPLC/Q-TOF-MS/MS analysis and network pharmacology was used to reveal the differences in the chemical components of AF and AFI that applied to their neuroprotective effects. The results indicated that 50 of the 188 compounds in AF and AFI may be bioactive, which may be related to their targeting of 108 targets such as XDH, GRIN2B, AKT1, PRKCG, CAPN1, CSNK2A1, G6PD. One hundred and forty-six important signaling pathways were implicated, including neurodegeneration (hsa05022), the Alzheimer’s disease pathway (hsa05010), the NF-kappa B signaling pathway (hsa04064), the HIF-1 signaling pathway (hsa04066), apoptosis (hsa04210), and the EGFR tyrosine kinase inhibitor resistance signaling pathway (hsa01521). These findings fully reflect the multi-component, multi-target, and multi-approach characteristics of TCM in disease treatment. This study shows that AF and AFI have great potential in neuroprotection, and their neuroprotective effects deserve further study.

In some network pharmacological studies, compounds are collected indiscriminately from databases; however, this can produce false-positive results. The method we applied in this research was built on the basis of experimentally identified components and corresponding targets, which will greatly reduce the prediction range and improve the accuracy of the prediction results. However, further pharmacological experiments are needed to verify its main biological components and related targets, so as to deeply understand the neuroprotective mechanism of AF and AFI, which will be the direction of our further research.

## Figures and Tables

**Table 1:** Compounds identified in AF and AFI by UNIFI software.

No.	Compound Name	Observed *m*/*z*	Mass Error (mDa)	Observed RT (min)	Adducts	AFI-CA D	AF-CAD	AFI-CA	AF-CA
1	7-Hydroxycoumarin	167.0119	1.5	1.01	-H_2_O+Na	✓	✓	✓	✓
2	Arginine	175.118	−1.0	1.05	+H	✓	✓	✓	✓
3	Isopimpinellin	251.0299	−1.6	1.09	-H_2_O+Na	✓			
4	Isoprenol	104.1062	−0.8	1.09	+NH_4_	✓	✓	✓	✓
5	Isomaltose	341.1088	−0.1	1.11	-H		✓		✓
6	Limonin	475.1763	3.6	1.12	-H_2_O+Na, +H	✓	✓	✓	✓
7	Farnesyl Acetate	287.196	−2.2	1.17	+Na	✓	✓	✓	✓
8	Heterodendrin	262.1276	−0.9	1.18	+H	✓		✓	
9	N-Methyl Proline	130.0852	−1.0	1.23	+H	✓	✓	✓	✓
10	Betonicine	160.096	−0.8	1.24	+H	✓	✓	✓	✓
11	Citric Acid	215.0155	−0.7	1.35	+Na	✓	✓	✓	✓
12	5-Hydroxymethyl Furaldehyde	127.0383	−0.7	1.35	+H	✓	✓	✓	✓
13	5-(Hydroxymethyl)Furan-3-Carbaldehyde	287.979	−0.7	1.36	-H_2_O+Na				✓
14	7-Hydroxy-6-Methoxy-Coumarin	193.0481	−1.4	1.39	+H	✓	✓	✓	
15	L-Synephrine Acetate	150.0913	0.0	1.42	-H_2_O+H	✓	✓	✓	✓
16	Dopamine	136.0746	−1.1	1.43	-H_2_O+H	✓	✓	✓	
17	L-Tyrosine	182.0808	−0.4	1.43	+H	✓		✓	✓
18	N-Methyltyramine	152.1065	−0.5	1.45	+H	✓	✓	✓	
19	Tyrosol	121.064	−0.8	1.45	-H_2_O+H	✓	✓	✓	✓
20	Dimethyl Anthranilate	166.0856	−0.7	1.55	+H	✓		✓	✓
21	Tyramine	120.0802	−0.6	1.56	-H_2_O+H	✓	✓	✓	✓
22	Citronellyl Acetate	203.1389	−1.7	1.62	-H_2_O+Na				✓
23	Salicylic Acid	137.024	−0.4	1.69	-H		✓		
24	Dehydrodieugenol	349.138	−3.1	1.73	+Na	✓	✓	✓	✓
25	Vanillin	153.0531	−1.5	1.74	+H	✓		✓	✓
26	Epigallocatechin	324.1077	0.0	1.76	+NH_4_			✓	
27	Rutin	611.1617	1.1	1.79	+H	✓	✓	✓	✓
28	Isocoumarin	147.0428	−1.2	1.93	+H		✓	✓	
29	Subaphylline	265.1542	−0.4	1.94	+H			✓	✓
30	Tryptophan	205.097	−0.1	2.00	+H	✓	✓	✓	✓
31	Geniposide	389.1408	−3.4	2.05	+H			✓	
32	Palmidin A	493.1303	2.1	2.05	-H_2_O+H	✓	✓		
33	4-Hydroxy-3-Methoxystrychnine	195.0659	−0.4	2.06	+HCOO			✓	
34	Caffetannic Acid	355.1007	−1.6	2.10	+H, +Na	✓	✓		
35	Ayapanin	177.0538	−0.8	2.12	+H				✓
36	Scolymoside	595.1667	1.0	2.20	+H	✓		✓	
37	Vicenin	595.1665	0.8	2.23	+H, -H_2_O+H	✓	✓	✓	✓
38	5,7-Dihydroxychromone 7-rutinoside	487.1441	−0.5	2.24	+H	✓	✓	✓	
39	Ferulic Acid	177.0537	−0.9	2.27	-H_2_O+H	✓	✓	✓	✓
40	Hyperoside	465.1027	0.0	2.31	+H	✓		✓	
41	Chrysophanol-1-O-β-gentiobioside	623.1598	−1.9	2.35	+HCOO, -H				✓
42	Benzoic acid	105.0325	−1.0	2.39	-H_2_O+H	✓	✓	✓	
43	Isorhamnetin-3-Rutinoside	625.1767	0.4	2.43	+H	✓	✓	✓	✓
44	Phenethylamine	144.079	0.7	2.55	+Na		✓		
45	Naringenin-4’-Glucoside-7-Rutinoside	765.2212	0.0	2.78	+Na	✓	✓	✓	✓
46	(+/−)-Naringenin	273.0752	−0.6	2.78	+H	✓	✓	✓	✓
47	Narirutin	581.1867	0.2	2.80	+H	✓		✓	✓
48	Phenylacetic acid	135.0446	−0.5	2.81	-H	✓		✓	
49	Salipurposide	435.1273	−1.3	2.82	+H	✓	✓	✓	✓
50	Methyl Chlorogenate	391.0965	−3.5	2.82	+Na	✓	✓	✓	✓
51	Eufin	123.0428	1.2	2.94	-H_2_O+Na			✓	
52	Cinaroside	449.1068	−1.0	2.94	+H, -H_2_O+H	✓			
53	Testosterone	293.1848	−2.8	2.94	-H_2_O+Na			✓	✓
54	Naringenin-7-O-Glucuronide	431.0937	−3.6	2.99	-H_2_O+H		✓		
55	2-Hydroxy-6-Methoxybenzoic Acid	151.0379	−1.1	3.10	-H_2_O+H, +H	✓	✓	✓	✓
56	Eriodictyol-7-Glucoside	473.1056	0.2	3.27	+Na	✓	✓	✓	✓
57	Coumarin	191.0345	−0.5	3.49	+HCOO	✓			
58	Vitamin B	442.1463	−0.7	3.64	+H	✓			
59	5,7-Dihydroxychromone	179.0328	−1.1	3.75	+H	✓		✓	✓
60	Butylidenephthalide	189.0897	−1.3	3.79	+H	✓	✓	✓	
61	Helenalin	263.1256	−2.2	3.88	+H	✓	✓	✓	✓
62	Emodin 8-glucoside	433.1119	−1.0	3.90	+H	✓	✓		
63	Kaempferol	287.0545	−0.5	4.15	+H	✓			
64	Genioisidic Acid	379.0991	−0.9	4.23	-H_2_O+Na		✓		
65	Eriodictuol	289.0688	−1.8	4.23	+H	✓	✓	✓	✓
66	Ombuin	331.0805	−0.8	4.24	+H, -H_2_O+H	✓			
67	Chrysophanein	417.1178	−0.2	4.24	+H	✓	✓	✓	✓
68	Lonicerin	595.1643	−1.4	4.28	+H	✓	✓	✓	✓
69	natsudaidain	419.131	−2.6	4.53	+H	✓	✓	✓	✓
70	Caffeic Acid	163.0376	−1.4	4.62	-H_2_O+H	✓	✓	✓	✓
71	Oleuropein	523.1775	−3.5	4.68	-H_2_O+H		✓	✓	✓
72	Hesperetin-7-O-β-D-Glucoside	487.1202	−0.9	4.82	+Na	✓	✓	✓	✓
73	Hesperidin Methyl Chalcone	625.2081	−4.6	4.85	+H		✓	✓	✓
74	3,4,7-Trimethoxycoumarin	237.0745	−1.3	4.94	+H	✓	✓	✓	✓
75	Narirutin-isomer	581.1862	−0.3	5.02	+H, +Na		✓		
76	Curculigoside	449.1429	−1.3	5.20	-H_2_O+H	✓	✓	✓	✓
77	Homoeriodictyol	303.0846	−1.7	5.22	+H	✓	✓	✓	✓
78	Chryso-Obtusin Glucoside	565.1554	−0.9	5.24	+HCOO	✓	✓	✓	✓
79	Rhoifolin	579.1714	0.5	5.34	+H, +Na	✓	✓	✓	✓
80	Eriocitrin	579.1701	−0.8	5.39	-H_2_O+H	✓	✓	✓	✓
81	Meranzin Hydrate	261.1109	−1.2	5.50	-H_2_O+H	✓	✓	✓	✓
82	Paeonioflorin	463.1566	−3.2	5.52	-H_2_O+H	✓	✓	✓	✓
83	Gallic Acid	153.0171	−1.1	5.60	-H_2_O+H	✓	✓	✓	✓
84	Physcion-8-O-Beta-D-Gentiobioside	609.1801	−1.3	5.79	+H	✓		✓	✓
85	Diosmin	609.1818	0.4	5.81	+H	✓	✓	✓	
86	Hesperetin-7-O-Neohesperidoside	633.1781	−0.9	6.02	+Na		✓		
87	Neohesperidin	633.1781	−0.9	6.02	+Na	✓			
88	Torachrysone	431.1337	2.5	6.03	+Na	✓	✓	✓	✓
89	Diosmetin	301.0697	−1.0	6.11	+H	✓	✓	✓	
90	Pinoresinol Dimethyl Ether	404.2054	−1.4	6.12	+NH_4_	✓	✓	✓	✓
91	Rubrofusarin-6-Β-Gentiobioside	595.1663	−0.6	6.31	-H			✓	✓
92	Hesperidins	633.18	1.0	6.65	+Na			✓	
93	Obtusin	345.0963	−0.6	6.79	+H	✓	✓	✓	✓
94	Coptisine	303.0894	0.4	7.08	-H_2_O+H	✓		✓	
95	Citromitin	449.1448	−0.6	7.12	+HCOO	✓	✓		
96	3-Tert-Butyladipic Acid	207.1001	1.0	7.66	-H_2_O+Na		✓		
97	Nomilinic acid Glucoside	717.2709	−2.0	7.76	-H_2_O+Na	✓	✓		✓
98	Deacetylnomilin	473.2162	−0.8	7.78	+H	✓	✓		
99	5,7,4’-Trimethoxyflavone	317.0769	−1.6	7.91	-H_2_O+Na	✓	✓	✓	✓
100	5,7-Dimethoxy Coumarin	189.0542	−0.4	7.92	-H_2_O+H	✓	✓	✓	✓
101	Dl-3-Phenyllactic Acid	189.0535	1.3	7.93	+Na	✓	✓	✓	✓
102	Seselin	227.0705	−0.8	7.94	-H		✓		
103	Resveratrol	227.0707	−0.7	7.94	-H	✓			
104	Salireposide	451.1231	−1.5	8.20	+HCOO		✓		✓
105	Meranzin	261.1111	−1.0	8.23	+H	✓	✓	✓	
106	Naringin	581.1859	−0.6	8.48	+H	✓	✓	✓	✓
107	Terpinyl Acetate	241.1441	−0.5	8.80	+HCOO		✓		✓
108	Eucommioside	385.1277	0.7	9.54	+Cl			✓	
109	6-O-Benzoylphlorigidoside B	551.1747	−1.2	9.79	-H_2_O+H	✓			
110	Obacunone	455.205	−1.4	9.84	+H	✓		✓	
111	Xanthotoxol	201.0183	−1.0	10.37	-H	✓	✓	✓	✓
112	Kaempferol-3-Arabofuranoside	441.0769	−2.3	10.56	+Na	✓	✓	✓	
113	Novobiocin	639.1925	0.0	10.66	-H_2_O+Na			✓	✓
114	Luteolin	285.04	−0.4	10.80	-H	✓			
115	Eucommin A	573.1938	−0.4	10.93	+Na	✓	✓	✓	✓
116	Citrusin B	573.1932	−1.1	10.94	-H_2_O+Na	✓		✓	
117	(+)-Threo-Guaiacylglycerol	219.0644	1.7	11.23	-H_2_O+Na				
118	Genipingentiobioside	585.1607	1.5	11.30	+Cl	✓			
119	Didymin	595.2036	1.4	11.35	+H, +Na	✓	✓	✓	✓
120	Isosakuranetin	287.0915	0.1	11.36	+H	✓	✓	✓	✓
121	Pectolinarin	623.197	−0.1	11.40	+H	✓	✓	✓	✓
122	Emodin Anthrone	257.0797	−1.1	11.54	+H	✓	✓	✓	
123	Lignans	415.1381	−0.6	11.79	+H			✓	
124	3,3’,4’,5,6,7,8-heptamethoxyflavone	433.1491	−0.2	11.80	+H	✓	✓	✓	✓
125	Physcion	283.0598	−1.4	12.41	-H	✓		✓	
126	Apigenin	269.045	−0.5	12.81	-H	✓			
127	Genistein	269.0445	−1.1	12.84	-H	✓	✓		
128	IsoMeranzin	243.1011	−0.5	13.17	-H_2_O+H, +H, +Na	✓	✓	✓	✓
129	5,2’,6’-Trihydroxy-7,8-Dimethoxyflavone	329.0651	−1.6	13.22	-H	✓		✓	
130	Tangeretin	373.1281	−0.1	14.19	+H, +Na	✓	✓	✓	✓
131	Chrysoobtusin	357.0971	−0.9	14.27	-H				✓
132	Gardenin B	359.111	−1.6	14.29	+H	✓			
133	P-Cymene	135.1158	−1.1	14.31	+H			✓	
134	Coniferin	297.1477	−0.8	14.32	-H_2_O+H	✓		✓	✓
135	Isolimonic Acid	489.2118	−0.1	14.36	-H_2_O+H			✓	✓
136	Vitamin E	491.2274	−4.9	14.39	+H			✓	
137	Marmin	355.151	−0.6	14.58	+Na	✓	✓	✓	✓
138	7-Hydroxyl-3,5,6,3′,4′-Pentamethoxyflavone	389.1222	−0.9	14.60	+H	✓	✓	✓	✓
139	Majudin	217.0487	−0.8	14.64	+H	✓	✓		✓
140	7-Methoxy-5-Prenyloxycoumarin	283.0932	−0.9	14.69	+Na				✓
141	5,2’,5’-Trihydroxy-6,7,8-Trimethoxyflavone	359.0775	0.2	14.75	-H	✓			
142	Gardenin A	419.132	−1.7	14.87	+H	✓	✓	✓	✓
143	Columbianadin	329.1352	−3.1	15.02	+H			✓	
144	Cucurbic Acid	211.1332	−0.7	15.04	-H				✓
145	Isosinensetin	373.1262	−2.0	15.25	+H	✓			
146	Sinensetin	373.1267	−1.5	15.26	+H, +Na		✓	✓	✓
147	Obacunoic Acid	473.2157	−1.3	15.28	+H	✓	✓	✓	✓
148	3,5,6-Trihydroxy-7,4’-Dimethoxyflavone	313.07	−0.7	15.48	-H_2_O+H			✓	✓
149	Javanicin	313.0693	1.1	15.48	+Na	✓			
150	4’,5,7,8-Tetramethoxyflavone	343.1172	−0.5	15.55	+H, +Na	✓	✓	✓	✓
151	Elemicin	231.1007	1.6	15.75	+Na	✓	✓		
152	Balanophonin	401.1233	−0.9	15.81	+HCOO				✓
153	Isolimonicacid 16->17-Lactone	471.2008	−0.6	15.92	-H_2_O+H, +H	✓	✓	✓	✓
154	Nobiletin	403.1412	2.5	16.40	+H	✓	✓	✓	✓
155	Thaliglucinone	388.1136	−2.0	16.42	+Na	✓	✓	✓	✓
156	Eupatoretin	373.0931	0.2	16.60	-H	✓			
157	Cassiaside	403.1016	−1.8	16.65	-H	✓			
158	Nomilinicacid	515.2277	0.2	16.96	-H_2_O+H	✓	✓	✓	✓
159	Nomilin	515.2261	−1.5	16.98	+H	✓			✓
160	Palmitic Acid	274.2735	−0.5	17.29	+NH4	✓	✓	✓	✓
161	Caffeic Acid Dimethyl Ether	191.0693	−0.9	17.39	-H_2_O+H				✓
162	3,5,6-Trihydroxy-7,3’,4’-Trimethoxyflavone	343.0804	−0.8	17.70	-H_2_O+H	✓	✓	✓	✓
163	Vomifoliol	247.1317	1.2	18.86	+Na				✓
164	2,4,4-Trimethyl-3-(3-Oxobutyl) Cyclohex-2-Enone	209.152	−1.6	18.89	+H	✓	✓	✓	✓
165	Tauremisin	265.1423	−1.1	18.90	+H, -H_2_O+H	✓		✓	✓
166	Dodec-2-Enal	200.1996	−1.3	18.94	+NH_4_	✓	✓	✓	✓
167	Phytosphingosine	318.2987	−1.6	20.36	+H	✓	✓	✓	✓
168	L-Leucine	130.0867	−0.6	20.57	-H	✓	✓	✓	✓
169	Aurapten	297.1522	2.6	20.80	-H	✓	✓	✓	✓
170	Thalcimine	619.2839	3.7	21.01	-H_2_O+H			✓	
171	Dodecanoic Acid	297.1523	0.4	21.04	+HCOO	✓	✓	✓	✓
172	Magnograndiolide	265.147	2.5	21.24	-H	✓	✓		
173	Isotetrandrine	640.3442	6.1	21.29	+NH_4_	✓		✓	
174	Palmitoleic Acid	277.215	1.2	21.38	+Na	✓	✓	✓	
175	Zoomaric Acid	277.2152	1.4	21.42	+Na				✓
176	Methyl Palmitate	315.2523	−1.8	21.45	+HCOO	✓	✓	✓	
177	Civetone	295.2277	−0.1	23.51	+HCOO	✓	✓	✓	✓
178	1-Palmitoyl-Sn-Glycero-3-Phosphocholine	496.3394	−0.3	24.01	+H	✓		✓	✓
179	Ochrolifuanine A	483.2731	−3.5	24.62	+HCOO	✓	✓		
180	Phthalic acid	149.0222	−1.1	25.28	-H_2_O+H	✓	✓	✓	✓
181	Diisobutyl phthalate	279.1582	−0.9	25.28	+H	✓		✓	✓
182	Monopalmitin	353.2665	0.2	26.50	+Na	✓	✓	✓	✓
183	Aplotaxene	277.2166	−0.7	27.71	+HCOO	✓	✓	✓	
184	Magnoflorine	377.1413	1.3	27.72	+Cl	✓			
185	Β-Sitosterol	397.3823	−0.6	28.02	-H_2_O+H				✓
186	(3R)-3-Methylpentanal	123.078	0.0	28.81	+Na	✓		✓	
187	Linoleic	263.2364	−0.6	29.19	-H_2_O+H, +NH_4_	✓	✓	✓	✓
188	β-Ecdysterone	481.313	−3.0	29.64	+H			✓	✓

**Table 2:** Neuroprotective candidate compounds in AF and AFI.

No.	Compound Name	AFI-CAD	AF-CAD	AFI-CA	AF-CA	No.	Compound Name	AFI-CAD	AF-CAD	AFI-CA	AF-CA
1	7-Hydroxycoumarin	✓	✓	✓	✓	60	Butylidenephthalide	✓	✓	✓	
2	Arginine	✓	✓	✓	✓	63	Kaempferol	✓			
3	Isopimpinellin	✓				65	Eriodictuol	✓	✓	✓	✓
5	Isomaltose		✓		✓	67	Chrysophanein	✓	✓	✓	✓
6	Limonin	✓	✓	✓	✓	74	3,4,7-Trimethoxycoumarin	✓	✓	✓	✓
7	Farnesyl Acetate	✓	✓	✓	✓	82	Paeonioflorin	✓	✓	✓	✓
8	Heterodendrin	✓		✓		88	Torachrysone	✓	✓	✓	✓
9	N-Methyl Proline	✓	✓	✓	✓	98	Deacetylnomilin	✓	✓		
11	Citric Acid	✓	✓	✓	✓	100	5,7-Dimethoxy Coumarin	✓	✓	✓	✓
14	7-Hydroxy-6-Methoxy-Coumarin	✓	✓	✓		101	Dl-3-Phenyllactic Acid	✓	✓	✓	✓
15	L-Synephrine Acetate	✓	✓	✓	✓	102	Seselin		✓		
16	Dopamine	✓	✓	✓		123	Lignans			✓	
20	Dimethyl Anthranilate	✓		✓	✓	125	Physcion	✓		✓	
22	Citronellyl Acetate				✓	127	Genistein	✓	✓		
23	Salicylic Acid		✓			130	Tangeretin	✓	✓	✓	✓
24	Dehydrodieugenol	✓	✓	✓	✓	140	7-Methoxy-5-Prenyloxycoumarin				✓
29	Subaphylline			✓	✓	152	Balanophonin				✓
32	Palmidin A	✓	✓			155	Thaliglucinone	✓	✓	✓	✓
34	Caffetannic Acid	✓	✓			160	Palmitic Acid	✓	✓	✓	✓
35	Ayapanin				✓	161	Caffeic Acid Dimethyl Ether				✓
44	Phenethylamine		✓			165	Tauremisin	✓		✓	✓
45	Naringenin-4’-Glucoside-7-Rutinoside	✓	✓	✓	✓	166	Dodec-2-Enal	✓	✓	✓	✓
46	(+/−)-Naringenin	✓	✓	✓	✓	168	L-Leucine	✓	✓	✓	✓
53	Testosterone			✓	✓	169	Aurapten	✓	✓	✓	✓
55	2-Hydroxy-6-Methoxybenzoic Acid	✓	✓	✓	✓	176	Methyl Palmitate	✓	✓	✓	

**Table 3:** The potential neuroprotective pharmacological targets of AF and AFI.

Excitotoxic				Antioxidant	
XDH	GRIN2B	APP	IL1B	CSNK2A1	G6PD
AKT1	PRKCG	PRKCA	CAPN1	NFKB1	FABP1
DAO	GRM2	MAPK10	SLC8A1	STAT3	NR1I3
GSR	ADORA2A	TP53	SLC1A1	CASP3	NR1I2
PARP1	GAPDH	PPARG	GRIK1	MAPK14	PPARA
SNCA	HSPA8	PLA2G2A	GRIA2	VCP	IL6
ACHE	SLC1A2	GLUL	BIRC3	BCL2	ICAM1
NOS2	CHRNA7	MAPT	BIRC2	CTSB	VCAM1
NOS1	PTGS2	PIK3CG	GRIN2A	NR1H4	HMOX1
JAK2	SRC	CDK5		PTGS1	ODC1
VEGFA	GRIN1	TGFB1		ALB	CREBBP
FGF2	TH	GRK2		NQO1	PGD
DRD2	HTT	XIAP		EP300	SOAT1
FOLH1	GRM5	TGM2		NOX4	HDAC3
OPRM1	CYP19A1	NTRK3		MPO	PLA2G6
MAPK1	GRIA4	HDAC9		CSNK2A2	PON1
TNF	DAPK1	PLAT		NFE2L2	CXCR3
BCL2L1	RPS6KA5	SLC1A3		ABCC1	SIRT3
KCNJ5	MAPK8	NTRK2		CXCL8	NR0B2
CNR1	GRIA1	PSEN1			GSTA1

## Data Availability

Data is contained within the article and Supplementary Materials.

## Supplementary Materials

The following supporting information can be downloaded at: https://www.mdpi.com/article/10.3390/ph17020239/s1, Table S1: Chemical compounds contained in citrus traditional Chinese medicine, Table S2: The entire list of targets of each compound, Table S3 1052 AFI- or AF-associated targets, Table S4: Excitotoxicity-associated targets, Table S5: Antioxidant-associated targets, Table S6: The Candidate Compounds and Potential Targets, Table S7: Information about the biological processes, Table S8: Information about the signaling pathways.
